# Are Female Sex Workers Able to Negotiate Condom Use with Male Clients? The Case of Mobile FSWs in Four High HIV Prevalence States of India

**DOI:** 10.1371/journal.pone.0068043

**Published:** 2013-06-28

**Authors:** Shalini Bharat, Bidhubhusan Mahapatra, Suchismita Roy, Niranjan Saggurti

**Affiliations:** 1 School of Health Systems Studies, Tata Institute of Social Sciences, Mumbai, India; 2 HIV & AIDS Program, Population Council, New Delhi, India; Indiana University and Moi University, United States of America

## Abstract

**Introduction:**

Condom promotion among female sex workers (FSWs) is a key intervention in India’s National AIDS Control Program. However, there is limited understanding of how FSWs negotiate condom use with male clients, particularly in the context of their mobility for sex work. The objective of this study is to examine the factors associated with the mobile FSWs’ ability to refuse unsafe sex and successfully negotiate condom use with unwilling male clients.

**Methods:**

Data for 5498 mobile FSWs from a cross-sectional survey conducted in 22 districts of four states in southern India were analyzed. Questions assessed FSWs’ ability to refuse clients unprotected sex, convince unwilling clients for condom use and negotiate condom use in a new location. Logistic regression models were constructed to examine the association between socio-demographics, economic vulnerability, sex work practice, and program exposure and condom negotiation ability.

**Results:**

A majority of FSWs (60%) reported the ability to refuse clients for unprotected sex, but less than one-fifth reported the ability to successfully convince an unwilling client to use a condom or to negotiate condom use in a new site. Younger and older mobile FSWs compared to those who were in the middle age group, those with longer sex work experience, with an income source other than sex work, with program exposure and who purchased condoms for use, reported the ability to refuse unprotected sex, to successfully negotiate condom use with unwilling clients and to do so at new sites.

**Conclusion:**

FSWs need to be empowered to not only refuse unprotected sex but also to be able to motivate and convince unwilling clients for condom use, including those in new locations. In addition to focusing on condom promotion, interventions must address the factors that impact FSWs’ ability to negotiate condom use.

## Introduction

Sex work is an age-old institution in India [1]. Contemporary sex work is a mix of the traditional *devadasi* system practiced in some parts of Karnataka [2], [3], largely brothel based as reported in West Bengal [4], predominantly street, lodge and home based in states of Tamil Nadu, and Andhra Pradesh [5], [6], and a combination of brothel, street, bars and home based in Maharashtra [7]. A majority of FSWs enter the profession at young age, are illiterate, from rural areas, and support young children, aged parents, and non-earning partners [8]. Many are pushed into the profession while some enter it out of economic compulsions and lack of skills to earn a living [9], [10]. Recent studies suggest a complex typology of FSWs in terms of the sites of solicitation (street, brothel, bars) and sites of sex work (brothel, lodge, home), and mobility patterns, with important implications for HIV interventions [11]. Sex work is highly stigmatized in India with female sex workers (FSWs) routinely discriminated in society and in public service institutions [4]. Violence and abuse by intimate partners, brothel owners, pimps and middle-men, and the police, are frequent occurrences in the lives of FSWs accentuating their overall vulnerability, particularly to sexual infections including HIV [12].

FSWs have been a key target population for HIV prevention from the beginning of India’s national response to HIV/AIDS [13]. Successive phases of India’s National AIDS Control Program (NACP I, 1992–1999; NACP II, 2000–2006, NACP III, 2007–2012) have focused on this key group, expanding and significantly scaling up activities and budget, as part of the targeted intervention approach among most–at-risk communities. This sustained focus has resulted in a reduction of national HIV prevalence among sex workers by more than 50% between 2003 and 2008 [13]. A range of interventions, both structural [12], [14]–[17] and behavioral [18], [19], are identified to account for this success. Promoting condom use with male clients is an integral component of most interventions among FSWs in the country. However, condom use with clients depends on the ability of FSWs to successfully negotiate its use with them. In the context of developing countries, women in general lack power to negotiate condom use with their male partners. Negotiating for safety in sexual matters is seen as violating cultural values and norms that dictate that women must be subservient to male sexual desire and demands [20]–[23]. FSWs lack power not only on account of being women but also due to being in an unequal relationship with male clients who are a source of income and thereby sustenance for them. Some studies report that FSWs in general lack the skills, ability and power to negotiate condom use with clients [24]–[26] and that power to negotiate is influenced by environmental-structural factors more than individual factors [14], [27], [28]. A few other studies indicate the importance of both individual factors (alcohol and substance use, depression) and environmental-structural factors (being trafficked into sex work, need to make more money, poor condom availability, being part of an organization of sex workers) in differentiating FSWs who usually negotiate condom use with clients from those who generally do not [29], [30].

Although condom promotion is a major approach in HIV prevention work among FSWs in India and increased condom use is reported over the three NACP phases [5], [6] there is limited understanding of how FSWs negotiate condom use with their male clients. Indeed, the FSW- client interaction for condom use is largely unexplored in the Indian context. Three issues are important to explore: a) are FSWs able to bring condom use into the picture/discuss condom use when they meet clients, b) are FSWs able to stand their ground/insist on condom use when clients refuse to use condoms, particularly if they are offered additional payment, and c) are FSWs able to convince unwilling clients to use condoms? These issues assume importance in the light of findings that almost one-third of FSWs either use condoms inconsistently or not at all [31], [32]. In the global literature, condom negotiation by FSWs with their clients is conceptualized in four different ways: “asking clients to use a condom”, “refusing to have sex if the client is not using a condom”, “refusing to have sex for more money if a client is not willing to use a condom” and “convincing an unwilling client to use a condom” [27], [29], [33], [34]. Most condom promotion interventions teach FSWs about negotiating condom use with clients (for example, “saying no” to sex without condoms, and ways to make condom use interesting) but empowering them to refuse clients who are unwilling to use condoms needs structural inputs (for example, adopting a condom use rule at the workplace). Such policy-level inputs influence condom negotiation intention as well as ability [27], [29]. While the frequency of condom use is assessed in surveys, few studies assess FSWs’ self-efficacy in negotiating condom use, which is the self-perceived ability to refuse sex without condoms or to successfully insist on condom use with clients. In the context of mobile FSWs, who are more vulnerable to HIV infection [32], the newness of sex work sites and environments can further challenge condom negotiation with clients. This paper focuses on two major aspects of condom negotiation among mobile FSWs operating in the four high HIV prevalence states in southern India. Specifically, the paper seeks to study the factors associated with mobile FSWs’ ability to: (i) refuse to have unsafe sex with clients, and (ii) successfully negotiate condom use with unwilling clients.

## Methods

### Research Design

This analysis is based on data from a cross-sectional survey conducted between September 2007 and July 2008 among FSWs in 22 districts of four states of India (Andhra Pradesh, Maharashtra, Tamil Nadu and Karnataka) [35]–[38]. These four states were identified as high HIV prevalence states by the National AIDS Control Organization (NACO) in 2005, while the districts were identified using independent mapping and enumeration data on FSWs collected by the State AIDS Control Societies (SACS) as well as Avahan (the India AIDS Initiative of the Bill & Melinda Gates Foundation). A two-stage sampling procedure was used to select FSWs from both brothel and non-brothel sites. In the first stage, small and large sex worker solicitation sites, including brothels and open solicitation sites such as highways, market areas and railway stations, were identified and mapped. Next, all the small and large solicitation areas were combined or divided into clusters, where each cluster consisted of 500 FSWs. Three such clusters from each district were randomly selected and FSWs were systematically sampled from both the open solicitation sites and the brothel sites to obtain a minimum of 1,500 eligible participants per state. Eligibility criteria for the study were: FSWs who were above the age of 18, who had moved to a minimum of two places for sex work in the last two years, and at least one of the moves was across the district. Data was collected through face-to face-interviews by trained multilingual interviewers using an interview schedule.

Ethical approval for the study was obtained from the Institutional Review Boards of the Population Council and the University of Manitoba, Canada. Participants were informed of the study objectives and their oral consent was obtained. Of the total eligible FSWs (5611), 113 were excluded: 15 were below 18 years, 21 refused to participate, 51 withdrew from the interview midway, and 26 were disqualified due to lack of socio-economic information. The sample available for final analysis totaled 5498 FSWs.

### Measures

Interviewers asked single item questions on FSWs’ socio-demographic characteristics, duration of engagement in sex work, income sources other than sex work, debt status, exposure to HIV prevention programs and experience of violence. Condom negotiation was assessed using three questions. Two questions explored FSWs’ ability to negotiate with clients who were not willing to use condoms. Two scenarios were created for such unwilling clients through the questions asked. The first question was: “Have there been times when a man refused to use condoms and you agreed to have sex without a condom?” A “No” answer was treated as FSWs’ ability to refuse a client for unprotected sex. The second question was: “Have there been times when a man refused to use a condom but you convinced him to use it?” A “Yes” answer was treated as FSWs’ ability to successfully negotiate condom use with an unwilling client. The third question was designed to assess FSWs’ self-efficacy in asking clients at a new location to use condoms: “Is it difficult for you to negotiate condom use with some clients when you move to a new place?” The answer, “Not at all difficult” was inferred as high self-efficacy in condom negotiation in a new location. For analysis purposes, answers to first question were labeled “ability to refuse unsafe sex” while answers to other two questions were combined to form a composite score “ability to negotiate condom use”.

The ability to negotiate condom use and the ability to refuse unsafe sex were analyzed in relation to independent variables, such as, socio-demographics (current age, education, marital status, place of residence); economic vulnerability assessed in terms of being in debt (measured using a single item question on whether FSW had any financial debt at the time of survey), under contract (with any sex workers’ agent), income sources other than sex work; individual risk, such as alcohol use prior to sex and experience of violence; structural factors, such as condom availability; sex work features, such as duration of engagement in sex work, freedom to choose sex work location and type of sex; and program exposure. Program exposure of FSWs was measured using information about their contacts with outreach workers from government, Avahan funded programs, and/or non-governmental organizations (NGOs) in the current place. Those indicating no contacts with outreach workers were considered not exposed to the HIV prevention program.

### Statistical Analyses

Percentages and summary measures like mean and standard deviations were calculated to present the profile of mobile FSWs. Two separate logistic regression models were constructed to examine the correlates of ability to negotiate condom use, and ability to refuse unsafe sex among FSWs. The following measures were included as controlling variables in the multivariate models: age, duration in sex work, education, marital status, place of residence, income other than sex work, current financial debt status, practicing sex work under contract, experience of any form of violence, alcohol consumption, decision related to place of sex, timing of sex work practice, exposure to HIV prevention program and purchasing condoms for own use. Most of these measures have been identified as important predictors of HIV risk behaviors and condom negotiation skills among FSWs in past research studies in India and elsewhere [29], [39]–[42]. Results from the logistic regression are presented in the form of adjusted odds ratios (AOR) and corresponding 95% confidence interval (CI). All the analyses were carried out using STATA version 12 (StataCorp., College Station, TX, USA).

## Results

The mean age of mobile FSWs in the sample was 30 years (standard deviation [SD]: 5.8 years) and the mean duration in sex work was 5.6 years (SD: 4.1 years) (Table 1). More than half the FSWs (52%) had education up to primary school and the majority lived in urban areas (85%). Just over half (52%) were formerly married, only one-third (34%) were currently married while 15% were never married. Almost half had a source of income other than sex work (44%), and a similar percentage of FSWs were in debt (45%); just one in 10 (10%) practiced sex work under contract. About three-fourths (70%) reported being exposed to HIV programs and 60% said they purchased condoms to use with clients.

**Table 1:** Profile of female sex workers in four states of India.

Background characteristics	% or Mean (SD) (N = 5498)
Age (years), mean (SD)	30.0 (5.8)
Duration in sex work (years), mean (SD)	5.6 (4.1)
**Education**	
Up to primary school	52.3
Secondary school or above	47.7
**Marital status**	
Formerly married	51.9
Never married	14.5
Currently married	33.6
% residing in urban area	85.4
% have income other than sex work	43.5
% reporting financial debt	45.4
% practicing sex work under contract	9.5
% experienced any form of violence	30.5
% consumed alcohol	61.9
% decided place of sex work independently or jointly with client	64.1
% practiced sex work within a fixed time	57.7
% exposed to HIV prevention program	71.2
% bought condom for own use	60.5

SD: Standard Deviation.

Around three-fifths (60%) of FSWs were able to refuse sex with a client who was not willing to use condoms, but comparatively fewer (17%) were able to successfully negotiate condom use with unwilling clients or be able to do so in new locations (Table 2). Younger (<24 years) and older (32+ years) FSWs were more likely to be able to negotiate condom use (<24 years vs. 24–31 years- 17% vs. 14%, AOR: 1.6, 95% CI: 1.2–2.0; 32+ years vs. 24–31 years- 22% vs. 14%, AOR: 1.3, 95% CI: 1.1–1.5) and refuse sex without a condom (<24 years vs. 24–31 years- 63% vs. 54%, AOR: 1.6, 95% CI: 1.3–2.0; 32+ years vs. 24–31 years- 69% vs. 54%, AOR: 1.2, 95% CI: 1.1–1.4) than those in the middle age group (24–31 years). Those educated beyond primary school were better at negotiating condom use (20% vs. 15%, AOR: 1.2, 95% CI: 1.1–1.5) and refusing unsafe sex (67% vs. 54%, AOR: 1.5, 95% CI: 1.3–1.7) than those educated up to primary school. Duration in sex work was positively associated with the ability at negotiating condom use and refusing unsafe sex. Compared to FSWs who were practicing sex work for less than 3 years, those practicing for 10 years or more were twice more likely to report ability to negotiate condom use (11% vs. 22%, AOR: 1.8, 95% CI: 1.3–2.3) and three times more likely to report ability to refuse unsafe sex (45% vs. 73%, AOR: 3.0, 95% CI: 2.4–3.7). The ability to negotiate for condom use was higher among FSWs who were currently married than never married (23% vs. 12%, AOR: 1.9, 95% CI: 1.4–2.5), were in debt than without debt (19% vs. 16%, AOR: 1.2, 95% CI: 1.0–1.4), who did not experience any violence than those who experienced violence (18% vs. 14%, AOR: 1.5, 95% CI: 1.2–1.8), did not consume alcohol than those who consumed alcohol (19% vs. 16%, AOR: 1.3, 95% CI: 1.1–1.5), did not practice sex work within a fixed time than those who practiced within a fixed time (21% vs. 15%, AOR: 1.7, 95% CI: 1.5–2.0), who were exposed to HIV prevention programs than those who had no exposure (19% vs. 13%, AOR: 1.5, 95% CI: 1.2–1.8) and those who purchased condoms than who did not (22% vs. 10%, AOR: 2.2, 95% CI: 1.8–2.6). Similarly, the odds of ability to refuse unsafe sex was higher among FSWs who were currently married (AOR: 1.5, 95% CI: 1.2–1.8), from rural areas (AOR: 1.2, 95% CI: 1.0–1.4), under debt (AOR: 1.3, 95% CI: 1.1–1.4), who did not experience violence (AOR: 1.7, 95% CI: 1.5–1.9), did not consume alcohol (AOR: 1.4, 95% CI: 1.2–1.6), decided the place of sex themselves or jointly with clients (AOR: 1.9, 95% CI: 1.6–2.1) and bought condoms for their use (AOR: 2.3, 95% CI: 2.0–2.6) as compared to their respective counterparts.

**Table 2:** Unadjusted percent and adjusted odds ratios predicting female sex workers’ ability to negotiate condom use and to refuse unsafe sex by socio-demographic and behavioral characteristics as the predictor variables, India.

	Ability to negotiate condom use		Ability to refuse unsafe sex	
	% (N)	Adjusted ORs (95% CI)^1^	% (N)	Adjusted ORs (95% CI)^1^
**Age (years)**				
<24	17.2 (528)	1.6 (1.2–2.0)	63.4 (528)	1.6 (1.3–2.0)
24–31	13.9 (3127)	Referent	53.6 (3127)	Referent
32+	22.4 (1843)	1.3 (1.1–1.5)	69.2 (1843)	1.2 (1.1–1.4)
**Duration in sex work (years)**				
<3	11.0 (1104)	Referent	44.7 (1104)	Referent
3–5	17.0 (2456)	1.6 (1.2–2.0)	56.4 (2456)	1.6 (1.4–1.9)
6–9	19.1 (1009)	1.6 (1.2–2.0)	72.8 (1009)	3.1 (2.5–3.8)
10+	22.3 (929)	1.8 (1.3–2.3)	72.6 (929)	3.0 (2.4–3.7)
**Education**				
Up to primary school	14.9 (2878)	Referent	53.5 (2878)	Referent
Secondary school or above	19.5 (2620)	1.2 (1.1–1.5)	66.7 (2620)	1.5 (1.3–1.7)
**Marital status**				
Formerly married	14.7 (2853)	1.3 (1.0–1.7)	51.7 (2853)	0.7 (0.6–0.9)
Never married	11.7 (795)	Referent	60.1 (795)	Referent
Currently married	23.2 (1848)	1.9 (1.4–2.5)	72.2 (1848)	1.5 (1.2–1.8)
**Place of residence**				
Urban	16.9 (4694)	Referent	58.5 (4694)	Referent
Rural	18.3 (804)	1.0 (0.8–1.2)	67.3 (804)	1.2 (1.0–1.4)
**Income other than sex work**				
No	15.5 (3107)	Referent	61.6 (3107)	Referent
Yes	19.1 (2391)	1.0 (0.9–1.2)	57.4 (2391)	0.7 (0.6–0.8)
**Currently in debt**				
No	15.5 (3003)	Referent	58.2 (3003)	Referent
Yes	19.0 (2495)	1.2 (1.0–1.4)	61.7 (2495)	1.3 (1.1–1.4)
**Currently under contract**				
No	17.5 (4974)	1.2 (0.9–1.6)	60.3 (4974)	0.8 (0.7–1.0)
Yes	13.0 (524)	Referent	55.0 (524)	Referent
**Experienced any form of violence**				
No	18.4 (3822)	1.5 (1.2–1.8)	63.2 (3822)	1.7 (1.5–1.9)
Yes	14.1 (1676)	Referent	52.0 (1676)	Referent
**Consumed alcohol**				
No	19.2 (2093)	1.3 (1.1–1.5)	66.0 (2093)	1.4 (1.2–1.6)
Yes	15.8 (3405)	Referent	55.9 (3405)	Referent
**Place of sex work decided by:**				
Client	17.1 (1976)	Referent	48.0 (1976)	Referent
Self or jointly with client	17.1 (3522)	0.9 (0.8–1.1)	66.4 (3522)	1.9 (1.6–2.1)
**Practiced sex work within a fixed time**				
No	20.6 (2326)	1.7 (1.5–2.0)	63.1 (2326)	1.2 (1.0–1.3)
Yes	14.5 (3172)	Referent	57.3 (3172)	Referent
**HIV prevention program exposure**				
No	13.1 (1581)	Referent	61.6 (1581)	Referent
Yes	18.7 (3917)	1.5 (1.2–1.8)	59.1 (3917)	0.9 (0.8–1.1)
**Bought condoms for own use**				
No	10.1 (2173)	Referent	45.6 (2173)	Referent
Yes	21.6 (3325)	2.2 (1.8–2.6)	69.1 (3325)	2.3 (2.0–2.6)
**Total**	**17.1 (5498)**		**59.8 (5498)**	

1 OR: Odds Ratio. CI: Confidence Interval.

Age-based analysis of FSWs (Table 3) showed that a significantly higher proportion of younger FSWs (<24 years) were never married, working under contract, and did not practice sex work within a fixed time compared to those in the middle (24–31 years) and older (32+ years) age groups. A higher proportion of FSWs in the older age group (32+ years) were formerly or currently married, had other sources of income along with income from sex work, were in debt, had exposure to HIV prevention programs and bought condoms for their use as compared to younger FSWs (<24 years).

**Table 3:** Female sex workers’ socio-demographic characteristics by age, India.

	Age (in years)			
	<24 (N = 528)	24–31 (N = 3127)	32+ (N = 1843)	P-value
Educated up to primary school	47.7	53.4	51.8	0.045
Never married	50.0	15.2	3.0	<0.001
Had income other than sex work	27.8	39.2	55.2	<0.001
Currently in debt	38.8	41.9	53.2	<0.001
Currently under contract	20.6	9.7	6.1	<0.001
Experienced any form of violence	30.1	29.3	32.6	0.048
Consumed alcohol	60.0	61.6	63.0	0.404
Place of sex work decided by clients	38.3	36.3	34.7	0.279
Did not practice sex work within a fixed time	49.8	41.6	41.3	0.001
Exposed to HIV prevention program	66.1	67.8	78.6	<0.001
Did not buy condoms for own use	41.5	48.1	24.4	<0.001

## Discussion

Several studies have documented the proportion of FSWs using condoms with clients; however, fewer studies report on the process of condom negotiation or the capability aspect, such as FSWs’ ability to successfully negotiate condom use when clients refuse. The analyses presented here provide critical evidence on how the FSW-client interaction on condom use proceeds and the outcomes of this interaction in different scenarios in the context of mobile FSWs. Findings show that the majority of mobile FSWs are able to decline sex with clients who refuse condom use, but comparatively fewer, less than one–fifth, are able to convince an unwilling client to use a condom or have the self-efficacy to negotiate condom use in new locations. It appears that turning down an unwilling client is the path of least resistance while convincing a client to use condoms or to do so in new work sites is seen as difficult or as not worth pursuing for fear of violence or abuse. A few other studies have also reported that FSWs lack the ability to persuade clients to use condoms [24], [43] resulting in non-use of condoms with clients [44]. Saggurti et al. [30] concluded that mobility has an impact on sex workers ability to practice safer sex mainly due to their lack of power to negotiate and use condoms. Interventions promoting “no sex without condoms” seem to be empowering FSWs to stand their ground in the face of a client not wanting to use condoms but are not empowering them enough to have the ability to insist the client uses one. Clearly, HIV prevention interventions need to go beyond empowering FSWs to turn down unwilling clients and build their skills in the art of persuasion.

The sampled mobile FSWs differed in their ability to negotiate condom use by key demographic, economic and other variables. Younger and older women were better than those in the middle age bracket (24–31 years) at both refusing sex without condoms and negotiating condom use which is similar to the findings of another study in Philippines [39]. The issue of demand and supply in the sex market determines the power of FSWs to enjoy some degree of autonomy. Being youthful gives the natural advantage to younger women, who can perhaps refuse unsafe sex in the knowledge that they are in higher demand than older women and there may not be a real dearth of clients for them; as a result, these younger women enjoy a structurally superior position to negotiate condom use with their clients. An earlier study reported younger FSWs were more likely than older FSWs to use condoms consistently, an indicator of their ability to successfully negotiate condom use [27]. In the case of older mobile FSWs, their ability to successfully negotiate condom use or to refuse unsafe sex may be due to their long experience of being in the sex trade, as a result of which they may have learned the skills to deal with clients on condom use. This is supported by the study findings since duration of being in sex work was positively associated with FSWs’ ability to refuse unprotected sex as well as their ability to insist on protected sex with unwilling clients, including those at new sites. Older FSWs were also significantly more exposed to HIV intervention programs, which may explain their better skills in condom use negotiation. Importantly, older FSWs (>31 years) had additional sources of income and hence could possibly afford to lose unwilling clients; in fact, they were the most successful in refusing unsafe sex as well as negotiating condom use with clients. The comparatively poorer negotiation ability of FSWs in the middle age bracket (24–31 years) can be explained in terms of their life stage as well as competition from younger FSWs. More than half the mobile FSWs in this age segment were formerly married while about one-third were currently married. Hence they can be expected to have young dependent children and consequently face more difficult economic conditions. This may compel them to agree to unsafe sex and discourage them from entering into the negotiation process with unwilling clients for fear of losing them. Having dependent children is reported to be associated with the practice of unsafe sex for extra money [45]. Additionally, about 40% of FSWs in our study were exclusively dependent on sex work for income. The analysis by Saggurti et al. [30] shows that the mean age of entry of mobile FSWs into sex work was 24.1 years, and that the majority who took up sex work for economic reasons and negative life conditions were in the age group 25–34 years, were previously married and had no source of income other than sex work. As FSWs advance in age, they face competition from their more youthful counterparts, which may also force them to compromise on safety by accepting clients who refuse to use condoms. Thus, for the youngest group of mobile FSWs their youth and attraction, while for the oldest group their experience and knowledge of the market, appear to give them a platform to negotiate condom use, whereas FSWs in the middle age segment are forced to compromise due to their higher economic burden, lack of additional income sources, and competition from younger FSWs. Interventions must therefore factor in the age, work experience, and economic situation of FSWs in HIV prevention activities. Indeed, a behavior change intervention with sex workers in Kenya found that those with more than four years of experience in sex work were nearly two-times more likely to attain positive behavior change in consistent condom use than their less experienced counterparts [46].

Having an additional income source and not being under contract possibly allowed mobile FSWs to be in a stronger position to refuse sex without condoms as well as to negotiate condom use even in new places. In both these situations FSWs can be expected to feel more in control over their interaction with clients because refusal may not have catastrophic consequences. However, contrary to what would be expected, the study shows that being in debt was not disempowering, which is surprising because FSWs who are in debt would be motivated to earn more and therefore more willing to compromise on safety by not refusing clients wanting unprotected sex. Elsewhere FSWs wanting to make more money are reported to be less likely to negotiate condom use [29]. This unexpected finding of the study needs further exploration. The practice of sex work under time constraints, that is, within a fixed duration, as reported by FSWs in the middle and older age groups, also negatively influences FSWs’ ability to negotiate condom use or refuse unprotected sex. Pressure of time could be due to hiring of a place for sex work for a fixed duration and/or the desire to entertain several clients in one night, resulting in insufficient time to negotiate with clients for condom use. A practice- related factor that facilitates condom negotiation is self-purchase of condoms. A study in Central America found that sex workers who self-purchased condoms reported higher consistent condom use than those who did not [47]. FSWs who buy condoms can be expected to possess condoms when they are approached by clients, and are certainly in a better position to discuss condoms and negotiate their use compared to those who depend on clients or on NGOs/CBOs to acquire them. Most of the older FSWs in the current study bought condoms for use. For mobile FSWs possessing condoms is even more critical as condom non-availability in new locations could mean compromising safety in sex work. Additionally, possessing condoms could be indicative of the seriousness of intentions to use them and hence of FSWs’ better ability to negotiate with clients. Other factors that affect FSWs’ ability to negotiate condom use are alcohol use prior to sex and experience of violence. This finding concurs with previous studies [29], [48]. Alcohol consumption and experience of violence are both high among mobile sex workers [2], [30] and are associated with high risk behavior. This strongly argues for the need to address alcohol-related abuse and violence among sex workers, particularly if they are mobile.

Program exposure clearly empowers FSWs to both refuse unprotected sex as well as to negotiate condom use with unwilling clients and in new locations. However, the effect of program exposure is much less pronounced on FSWs’ ability to persuade clients to engage in protected sex than on their ability to refuse clients for unprotected sex. As mentioned earlier, this underscores the need to build FSWs’ persuasive powers and their skills in negotiating condom use in new places they visit when mobile. Persuasion may be understood as the art of convincing the client on the benefits of condom use for safeguarding him, his spouse and children from sexual infections and/or his loved ones in general, from burden of ill-health. It requires the FSW to go beyond educating clients about condom benefits to, for instance, presenting a choice of condom brands, suggesting novel ways of using condoms, and offering to help with the act of putting on the condom during foreplay. Ghose et al. [16] report how FSWs from the Sonagachi brothel area in Kolkata, India, are able to successfully convince clients to use condoms after they are aroused as a result of foreplay. The FSWs are thus able to transform ‘condoms into performative and transactional devices’ and teach each other these strategies through ‘bridging practices’ such as peer education and other educational activities [16]. Role play has been found to be a particularly effective technique in improving persuasive skills and self-efficacy of sex workers in interacting with clients and negotiating for safer sex [49]. Role plays can thus be integrated into negotiation skills training workshops for FSWs together with education on benefits of condom use. For mobile FSWs such as those who participated in this study, role plays will have to address the additional challenge of dealing with clients in non-familiar and often non-supportive settings. A major challenge would be how to reach mobile FSWs who are highly dispersed and operate either individually or as small groups controlled by middlemen. Community mobilization and empowerment approaches are of limited use with mobile FSWs [50] requiring some innovative ways of reaching them such as, during festivals and religious fairs that draw mobile FSWs in large numbers [2]. Peer educators trained in role plays and persuasion skill building could be employed for reaching mobile FSWs during festivals and similar large gatherings. In addition to these, HIV prevention programs need to devise strategies tailored to the sex work settings. In venue based settings for example, structural interventions that engage brothel owners can create enabling environment for safe sex negotiation with clients [51] while in contexts of multiple settings of sex work, community mobilization activities can be more effective in increasing FSWs’ self-efficacy for condom use and negotiation power [9].

Although the findings of this analyses on the factors associated with condom use negotiation among FSWs have important programmatic implications, they must be interpreted in light of certain limitations. First, the study did not use a comparative research design to include non-mobile FSWs; hence the findings apply only to those FSWs who moved and not to the general community of sex workers. Also, it explains condom use in the context of mobility but does not explain mobility as a driver of poor condom negotiation ability, for instance, how mobility contributes to FSWs’ inability to persuade clients for condom use. Another limitation is that the questions used to measure condom negotiation did not explore FSWs’ behavior in the scenario when clients offered more money for unsafe sex. Future research must better explore the negotiation process, preferably using a mixed-methods study design. Condom negotiation is likely to be influenced by the type of client- occasional or regular; paying or non-paying- however, this paper did not include this dimension in the analysis.

Available evidence for this sub-group of FSWs [2], [30] highlights that mobility enhances their vulnerability, and a high proportion of FSWs in India are mobile either due to economic compulsions, increasing competition or to avoid being identified as sex workers. The research findings reported here suggest that current interventions need to address the economic vulnerability of mobile FSWs and strategize differently for them as they are less likely to access static services. Most importantly, this study highlights the importance of empowering mobile FSWs in the art of persuading clients to use condoms and not just in their ability to “say no” to clients who refuse to use condoms.
